# A Guide to an Effective Peer Review

**DOI:** 10.24908/pocusj.v10i02.20046

**Published:** 2025-11-17

**Authors:** Tanping Wong, Riya N. Soni, Casey Glass

**Affiliations:** 1 Division of Hospital Medicine, Weill Cornell Medicine, New York, NY, USA; 2 University of Texas Health San Antonio, San Antonio, Texas, USA; 3 Department of Emergency Medicine, Wake Forest University School of Medicine, Winston-Salem, NC, USA

**Keywords:** Peer review, Scientific publishing, Manuscript evaluation, Reviewer guidance, Critical appraisal

## Abstract

Peer review is a fundamental element of the modern scientific publishing process. It serves an important role in evaluating the quality of research and refining submitted manuscripts into accurate and impactful contributions to the existing scientific literature. Over the last two decades, opportunities for publication have skyrocketed, and the demand for peer reviewers has grown exponentially. Although the peer review process provides significant benefits, recruiting individuals as peer reviewers can be challenging. The most common obstacles include the time commitment needed to provide meaningful reviews and uncertainty about how to prepare a cohesive and beneficial peer review. This article offers prospective peer reviewers structured guidance to build confidence and enable them to perform effective reviews.

## Should I Review This Paper?

The first step in the peer review process is deciding to accept or reject an invitation. When considering a peer review opportunity, there are three key questions reviewers can ask to determine their suitability as a reviewer: 1) Do I have sufficient expertise in the area of interest? 2) Can I complete the peer review in the allotted time? and 3) Do I have any ethical conflicts of interest?

The role of peer reviewers is to provide insightful commentary and suggestions to the editor, drawing on their familiarity of the field and their assessment of the submitted manuscript. Prospective peer reviewers often feel they lack the expertise to participate in peer review and mistakenly believe they need to be exceptionally talented in their field to provide meaningful feedback on submissions. In reality, the primary skill to contribute to peer review is the ability to critically read scientific literature and recognize both the positive and negative traits inherent in any scientific submission. With a core understanding of subject knowledge, critical reading and reviewing skills can be cultivated with experience and strengthened through mentored peer review submissions.

It is not unusual for peer reviewers to be intimidated by the statistical methods used in many modern research projects. Many, if not all, academic journals will have a statistics editor available to review the appropriateness of statistical methods used in a submission. Reviewers may suggest or request additional review by the statistics editor when preparing a peer review. Expertise in statistics is not a requirement. One of the main benefits of participating as a peer reviewer is the chance to increase familiarity with the appropriateness of various statistical approaches in different research settings.

Delays in the peer review are a major hurdle in the timeline for academic publishing. Journals typically set specific deadlines for when to respond to the review request and when to submit reviews. A common challenge in the initial steps of the review process is that requests often go unanswered, leading to extended delays in the peer review process. If a reviewer cannot meet either deadline, it is best to notify the editors immediately and decline the peer review. The earlier the editors are informed that a prospective reviewer is unable to complete the review, the sooner they can consider other potential reviewers.

Additionally, potential reviewers must assess if they meet the ethical requirements for peer review. Several publication ethics committees (e.g., Committee on Publication Ethics and the International Committee of Medical Journal Editors) have listed ethical guidelines when considering and performing peer review. Generally, manuscripts should not directly compete with a reviewer's own academic research or projects. It is inappropriate to peer review for authors with whom reviewers have or have had a close personal or professional relationship. Unpublished manuscripts are confidential and should not be shared with others or integrated into reviewers' own work until the manuscript is formally published.

Serving as a peer reviewer is an invaluable experience with both professional and personal benefits. Reviewers gain exposure to a wide variety of study designs and statistical methods while exercising and strengthening critical appraisal skills. They can stay abreast of the latest research advancements, connect with editors and experts in their field, and foster relationships to promote academic recognition. Beyond enhancing scholarly aspects, peer review can provide a sense of personal satisfaction by allowing reviewers to directly contribute to shaping the direction of research.

## Reviewing the Manuscript

The goal of peer review is to determine if the manuscript aptly addresses the research question, to assess if the findings are accurate, and to place the research in the context of existing scientific literature. We recommend a two-step approach to reviewing a manuscript. The first step is to read the manuscript in full. The reviewer should review the work from a broad outlook, considering whether it offers new or insightful contributions to the field and whether there are major flaws that compromise the validity of the findings. A brief literature search at this stage may help contextualize the submission and assess its novelty. The second reading should involve a more detailed assessment that systematically evaluates each section (Table 1). Specific concerns should be clearly noted with line numbers or paragraph locations so that the authors can easily address them. Reviewers are recommended to limit commentary regarding grammar or style as these tasks are better served by the journal's copy editors.

**Table 1.** Review Guidelines by Manuscript Section

Section	Guiding Questions
Title	Does the title clearly convey the research question and content?
Abstract	Is the abstract concise and brief? Do the abstract methods include enough information to generally interpret the study's results? Do the abstract results align with the study's reported findings? Do the abstract conclusions align with the study's conclusions? Does the abstract avoid overstating the importance of the study?
Introduction	Is the introduction clear and focused? Is sufficient context provided to understand the rationale for the study? Does the introduction clearly present the study's research question, objective, and scope?
Methods	Is enough methodological detail provided to replicate the study? Are the statistical analyses appropriate and understandable? Do the reported statistics require review by the statistics editor? Does the study comply with ethical standards (e.g., informed patient consent, Institutional Review Board (IRB) approval, etc.)?
Results	Do the reported results address the research question and objective? Are any included tables and figures appropriately referenced within the text? Are table or figure legends clear and understandable?
Discussion	Are the results appropriately interpreted and contextualized within the existing knowledge of the topic? Are the limitations of the study appropriately stated?
Conclusion	Does the conclusion align with the research question stated in the introduction? Does the conclusion avoid overstating the importance of the results? Does the conclusion avoid expressing opinions and unsupported claims?
References	Is self-citation maintained at a reasonable amount? Are cited sources interpreted accurately? Are important and relevant references missing from citations?

## Writing and Submitting the Review

The purpose of peer review is to enhance the quality of journal submissions by ensuring that the information in a manuscript is accurate, clearly communicated, and contributes meaningfully to the field. It is not intended as an obstacle to publication; rather, reviews should be constructive and adopt a collegial tone. An effective review often begins with a concise summary of the manuscript, accompanied by a brief assessment of its novelty and significance within the existing literature (Table 2). This is followed by a discussion of any major issues, such as methodological limitations, ethical concerns, or questions regarding the contribution of the work. Finally, reviewers may highlight minor points that could improve clarity or readability, including ambiguous phrasing or inappropriate citations.

**Table 2.** Reviewer Evaluation Outline

Review Section	Tasks
Summary	Briefly describe your overall impression of the work Indicate the type of research study based on your reading of the manuscript (e.g., randomized controlled trial, retrospective chart review, etc.) Describe the relative importance and impact of this work
Major Concerns	Prioritize the key points that are most important for the authors and editor List any concerns that affect the validity of the paper Inadequate research design Insufficient evidence to support the conclusion Ethical issues (confidentiality, anonymity, consent) Lack of novelty or meaningful contribution
Minor Concerns	Unclear or ambiguous sentences Reference errors Suggestions for better labeling of figures, tables, or sections
Comments to the Authors	Provide specific advice to address major concerns Avoid editorializing beyond the scope of a peer review
Comments to the Editor	Provide recommendations on publication Provide a specific rationale for your publication recommendation

Since reviewer comments are generally shared with authors verbatim, it is important to frame feedback in a constructive and respectful manner. Recommendations about acceptance or rejection should not appear in this portion of the review, as such judgments are reserved for the confidential comments to the editor. These final comments, which may or may not be shared with authors depending on journal policy, serve as the appropriate place to provide an overall recommendation—whether acceptance, revision, or, in rare cases where the manuscript has unresolvable flaws, rejection.

## Conclusion

Engaging in peer review is an act of service to the scientific community that concurrently offers significant benefits to reviewers. As with most professional skills, proficiency as a peer reviewer develops through regular practice. Experience in reviewing has been shown to enhance critical appraisal abilities and can contribute to improvements in one's own research design, analysis, and academic writing.
